# Endoscopic Screening for Missed Lesions of Synchronous Multiple Early Gastric Cancer during Endoscopic Submucosal Dissection

**DOI:** 10.1155/2023/2824573

**Published:** 2023-04-05

**Authors:** Jiangnan Wan, Yi Fang, Haizhong Jiang, Bujiang Wang, Lei Xu, Chunjiu Hu, Honghui Chen, Xiaoyun Ding

**Affiliations:** ^1^Department of Gastroenterology of Ningbo First Hospital, Ningbo, Zhejiang, China; ^2^Department of Gastroenterology of Ningbo Yinzhou No. 2 Hospital, Ningbo, Zhejiang, China

## Abstract

**Aims:**

To evaluate the value of endoscopic screening during endoscopic submucosal dissection (ESD) in the detection of synchronous multiple early gastric cancer (SMEGC) and the risk factors for missed diagnosis of SMEGC.

**Methods:**

We conducted gastric endoscopic screening during ESD operation in 271 patients with early gastric cancer (EGC) referred for ESD, and endoscopic follow-up within 1 year after the operation. The detection and characteristics of SMEGC were analyzed in three stages: before ESD, during ESD operation, and within 1 year after ESD.

**Results:**

SMEGC was detected in 37 of 271 patients (13.6%). Among them, 21 patients with SMEGC (56.8%) were diagnosed before ESD, 9 (24.3%) were diagnosed with SMEGC by endoscopic screening during ESD operation, and 7 (18.9%) were found to have EGC lesions in the stomach during postoperative endoscopic follow-up within 1 year. The preoperative missed detection rate of SMEGC was 43.2%, and the rate of missed detection could be reduced by 24.3% (9/37) with endoscopic screening during ESD operation. Missed SMEGC lesions were more common in flat or depressed type and smaller in size than the lesions found before ESD. The presence of severe atrophic gastritis and age ≥60 years were significantly correlated with SMEGC (*P* < 0.05), while multivariate analysis showed that age ≥60 years was an independent risk factor (OR = 2.63, *P* < 0.05) for SMEGC.

**Conclusions:**

SMEGC lesions are apt to be missed endoscopically. Special attention should be paid to small, depressed, or flat lesions in detecting SMEGC, especially in elderly patients or (and) patients with severe atrophic gastritis. Endoscopic screening during ESD operation can effectively reduce the missed diagnosis rate of SMEGC.

## 1. Introduction

Gastric cancer is one of the most common malignancies worldwide. Early gastric cancer (EGC) is defined as gastric carcinoma limited to the mucosa or submucosa, regardless of lymph-node metastasis, which has a good prognosis with a 5-year survival rate over 90% [1]. Endoscopic submucosal dissection (ESD) has been accepted as a standard treatment for EGC with the minimal risk of lymph-node metastasis, which is a minimally invasive technique that preserves the entire stomach of EGC patient. Meanwhile, long-term prognosis of ESD for the patients with EGC meeting the indications had been confirmed to be similar compared with surgery [2].

With the recent advances in endoscopic examination, more EGCs are detected. Sometimes, two or more cancerous lesions can be found endoscopically in the patient with EGC, which is defined as synchronous multiple early gastric cancer (SMEGC). The prevalence of SMEGC was reported to be 4–15% among all EGCs [3–5], especially in East Asia, where the SMEGC detection rate is relatively high. SMEGC diagnosis is of great importance because SMEGC lesions are easy to be missed. If synchronous cancer lesions are overlooked, patients may miss the opportunity to be treated in the early stage. Therefore, detection of SMEGC lesions is mandatory for a good prognosis for EGC patients who receive ESD.

In this study, we evaluated endoscopic screening in the patients with EGCs referred for ESD. The endoscopic screening was performed again during ESD operation to observe whether there were missed SMEGC lesions in the stomach.

## 2. Materials and Methods

### 2.1. Patients

**Figure 1:**
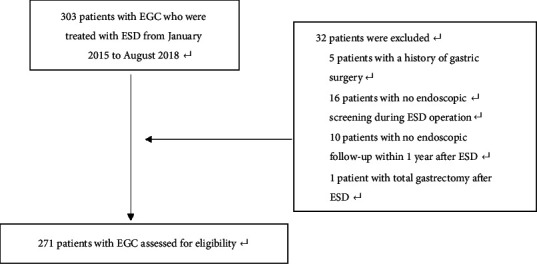
Patients selection.

A total of 303 consecutive patients diagnosed as EGC were treated with ESD in our single center from January 2015 to August 2018. The patients were excluded with a history of gastric surgery (5 patients), without endoscopic screening during ESD operation (16 patients), with no endoscopic follow-up within 1 year after the operation (10 patients), and with additional total gastrectomy after ESD (1 patient) (Figure 1). In 234 patients with solitary EGC, 10 cases received additional subtotal gastrectomy, while in 37 patients with SMEGC, 3 cases were referred for additional gastrectomy after ESD.

### 2.2. Definition

SMEGC is defined according to Moertel's standard [6] as follows: (1) each lesion is pathologically a malignancy; (2) all lesions are clearly separated by a microscopically normal gastric wall; and (3) there is no possibility that one of the lesions represents local extension or a metastatic tumor. Early cancerous lesions in the other sites of stomach are found within 1 year after ESD as SMEGC. Endoscopic screening was performed during the process of ESD. Before the operation began, the stomach was examined by endoscopy again to determine the presence of multiple lesions.

### 2.3. Diagnostic Criteria

The lesions and patients were classified according to the standards of the Japanese Gastric Cancer criteria.

Location: the stomach was divided into three parts according to the longitudinal axis: the upper third, middle third, and lower third.

Macroscopic classification: the lesions were classified into three types according to the macroscopic classification: elevated type (0-I, 0-IIa, 0-I + IIa, 0-IIa + IIb, and 0-IIa + IIc); flat type (0-IIb); and depressed type (0-IIc, 0-III, 0-IIc + IIa, and 0-III + IIa).

Histology: the lesions were classified into two different types according to the histological classification as follows: the differentiated type, which was divided into papillary adenocarcinoma and well or moderately differentiated tubular adenocarcinoma; and the undifferentiated type, which was divided into poorly differentiated tubular adenocarcinoma, signet-ring cell carcinoma, and mucinous adenocarcinoma.

Determination of main and minor lesions: according to the infiltration depth and maximum diameter of the lesions, the main lesions were evaluated by the worst pathological type (progression and/or size) of multiple lesions, and the main lesions represented the degree of disease.

Atrophic gastritis: endoscopic atrophic gastritis was classified according to the Kimura-Takemoto classification [7] as no gastritis, C-1 and C-2 (mild grade), C-3 and O-1 (moderate grade), or O-2 and O-3 (severe grade).

### 2.4. ESD Procedures

Each patient was anesthetized with general intravenous anesthesia. The gastric cavity was cleaned under endoscopy, and endoscopic screening was performed again (Olympus 260H, 290HQ, 260HZ, and 260J) to observe the presence of multiple lesions by a senior experienced endoscopist. ESD was performed with the 260J endoscope, and 1 mg epinephrine and 0.5 ml of methylene blue were added to 250 ml isotonic saline to prepare the solution used for submucosal injection. Marking dots were applied 3–5 mm outside the border of the lesions using electric coagulation. Then, the lesions were entirely removed by circumferential mucosa incision and submucosal dissection (hook knife, dual knife, IT knife). When active bleeding was observed, endoscopic hemostasis was performed either with the knife itself, hemostatic forceps, or hemostatic clips. Finally, specimens of the lesions were fixed and sent for pathological diagnosis.

### 2.5. Follow-Up

After ESD, patients were required to undergo endoscopic follow-up at the 3rd, 9th, and 21st months and once a year thereafter. During endoscopic follow-up, biopsies were performed for all suspected mucosal lesions.

### 2.6. Statistical Analysis

SPSS 23.0 software was used for statistical analysis. The statistical data were analyzed by Pearson's *x*^2^ test or Fisher's exact probability test, the measurement data were analyzed by *T*-test, and the independent risk factors were further screened by logistic regression multivariate analysis. *P*-values < 0.05 were considered statistically significant.

## 3. Results

### 3.1. Patient and Lesion Characteristics

A total of 271 patients were included in the study, 180 males and 91 females, aged 36–85 years, with an average age of 62.8 ± 8.3 years. Thirty-seven patients were diagnosed with SMEGC: 28 males and 9 females, aged 42–76 years, with an average age of 64.2 ± 6.9 years. A total of 78 lesions were found in 37 cases. All patients underwent endoscopy 1–4 times before ESD, with an average of 1.95 times. Endoscopic follow-up was conducted 1–2 times within 1 year after ESD, with the median endoscopic follow-up time of 283 days.

Among the 271 patients, 25 (9.2%) had a family history of gastric cancer. There were 77 (28.4%) and 98 (36.2%) patients with alcohol and tobacco intake histories, including 34 (12.6%) heavy drinkers and 74 (27.3%) heavy smokers. Eighty-nine patients (32.8%) were diagnosed with severe atrophic gastritis on endoscopy. *Helicobacter pylori* infection was diagnosed in 69 patients (25.5%). A total of 253 lesions were the differentiated type (93.4%), and 18 were the undifferentiated type (6.6%).

Thirty-seven (13.6%) patients were found to have SMEGC. Additionally, there were 78 lesions, including 33 cases of 2 lesions and 4 cases of 3 lesions. The average diameter of the lesions was 1.17 ± 1.12 cm. Eleven lesions were located in the upper 1/3 (14.1%), 22 lesions in the middle 1/3 (28.2%), and 45 lesions in the lower 1/3 (57.7%) of the stomach. There were 37 lesions (47.4%) of the elevated type, 7 lesions (9.0%) of the flat type, and 34 lesions (43.6%) of the depressed type. There were 75 lesions (96.2%) in 35 patients of the differentiated type and 3 lesions (3.9%) in 2 patients of the undifferentiated type. Four patients had 6 lesions (7.7%) infiltrating the muscular mucosa, and two patients had 2 lesions (2.6%) infiltrating the submucosa.

### 3.2. Predictive Risk Factors for SMEGC

As shown in Tables 1 and 2, the proportion of patients aged ≥60 years in the SMEGC group was higher than that in the solitary EGC group (83.8% vs. 64.5%, *P* < 0.05), and the proportion of patients with severe atrophic gastritis was also significantly higher than that in the solitary EGC group (48.6% vs. 30.3%, *P* < 0.05). In the SMEGC group, men accounted for 75.7% of patients, and those with smoking habits accounted for 48.6%, which were both higher than those in the single EGC group, but the differences were not statistically significant. Meanwhile, there were no significant differences in the family history of gastric cancer, drinking habits, *H. pylori* infection, or pathological types between the multiple groups and the single group. In multivariate analysis (Table 2), an age ≥60 years old (OR = 2.63, *P* < 0.05) was a statistically significant independent predictive factor for SMEGC.

### 3.3. SMEGC Detection at Different Endoscopic Screening Stages

As shown in Tables 3, 4, and 5, 37 patients were found to have SMEGC (13.6%) with a total of 78 lesions, including 33 cases with 2 lesions, 4 cases with 3 lesions; as well as 75 differentiated lesions in 35 cases, 3 undifferentiated lesions in 2 cases, 6 lesions infiltrating the muscular mucosa in 4 cases, and 2 lesions infiltrating the submucosa in 2 cases. Twenty-one patients with SMEGC were diagnosed before ESD, 19 patients with 2 lesions and 2 patients with 3 lesions. In 9 patients, missed multiple early cancerous lesions were detected by endoscopic screening during ESD operation, 8 patients with 2 lesions and 1 patient with 3 lesions. Within 1 year of postoperative follow-up, 7 cases were found to have EGC lesions, 6 cases with 1 lesion and 1 case with 2 lesions.

The preoperative detection rate of SMEGC was 56.8% (21/37), while the detection rate of SMEGC in intraoperative endoscopic screening was 24.3% (9/37), and that in postoperative follow-up within 1 year was 18.9% (7/37). The missed detection rate of SMEGC at the preoperative endoscopic screening was as high as 43.2% (16/37). Among them, 9 cases of multiple lesions were detected by endoscopic screening during the operation, which consisted of 56.3% (9/16) in the missed diagnosis cases and 24.3% (9/37) in the overall SMEGC cases.

### 3.4. Features of Missed SMEGC Lesions

A total of 17 lesions were missed preoperatively in 16 patients, among which 4 lesions were the elevated type, 5 lesions were the flat type, and 8 lesions were the depressed type. The average diameter of the missed lesions was 0.69 ± 0.43 cm. According to the site of the lesion, 3 lesions were located in the upper 1/3 of the stomach (17.7%), 5 lesions in the middle 1/3 of the stomach (29.4%), and 9 lesions in the lower 1/3 of the stomach (52.9%). A total of 9 missed lesions were detected by endoscopic screening during ESD operation, with an average diameter of 0.57 ± 0.17 cm with 2 lesions of the elevated type (22.2%), 1 lesion of the flat type (11.1%), 6 lesions of the depressed type (66.7%), 2 lesions in the middle third (22.2%), and 7 lesions in the lower third (77.8%). All the lesions were differentiated and intramucosal cancer. While 8 missed lesions in 7 patients were found on endoscopic follow-up within 1 year after ESD, the mean diameter of the lesions was 0.91 ± 0.67 cm with 2 lesions of elevated type (25%), 4 lesions of flat type (37.5%) in 3 patients, 2 lesions of depressed type (37.5%), 3 lesions in 2 patients in the upper 1/3 of the stomach (37.5%), 3 lesions in the middle 1/3 of the stomach (37.5%), and 2 lesions in the lower 1/3 of the stomach (25%). All 7 cases were intramucosal differentiated lesions.

As shown in Table 6, the percentage of missed lesions that were flat and depressed was significantly higher than that of the elevated type (76.5% vs. 23.5%, *P* < 0.05). Furthermore, the size of missed lesions was smaller (0.69 ± 0.43 cm vs. 1.30 ± 1.21 cm, *P* < 0.05). However, there was no significant difference between the location of missed foci and the type of differentiation.

## 4. Discussion

Due to the characteristics of EGCs, endoscopic detection and diagnosis of EGC lesions are still difficult, and missed diagnosis of EGC during gastroscopy is relatively common. Particularly for SMEGC lesions, the rate of missed diagnosis is much higher. Epidemiological studies show that SMEGC accounts for 4–15% of all EGCs [3–5]. In our study, SMEGC accounted for 13.6% of all ESD cases, suggesting that the incidence of SMEGC is not low and is worthy of more attention.

A few studies found that SMEGC frequently occurred in elderly male patients. Nitta et al. [8] investigated the clinicopathological differences between 94 cases of SMEGC and 285 cases of single EGC and found that the average age of the patients with single EGC was 62.4 years, while that of those with SMEGC was 66.1 years (*P* = 0.0009). The odds ratio was highest when subjects were divided into groups with a cutoff age of 65 years (OR = 2.19). The same conclusion was reached in the study of Jang et al. [9] in which an age ≥65 years was an independent risk factor for SMEGC (OR = 2.05, *P* = 0.012). The results of Nitta et al. also showed that a severe distribution of atrophic mucosa in the stomach and a severe degree of intestinal metaplasia were related to the occurrence of SMEGC. Multivariate analysis identified a severe degree of Intestinal metaplasia in the surrounding mucosa as a significant independent risk factor for SMEGC (OR = 2.75; *P* = 0.004). In another study, Lim et al. [10] analyzed synchronous EGC found during follow-up after EGC endoscopic resection and showed that age ≥65 years old, male sex, mucosal atrophy, and intestinal metaplasia were risk factors for SMEGC. Our study shows that an age ≥60 years and the presence of severe atrophic gastritis are predictive risk factors for SMEGC, and an age ≥60 years is an independent risk factor. SMEGC lesions were reported to generally take place in the middle and lower third of the stomach. In our study, 57.7% of the multiple lesions were located in the lower third of the stomach, which was probably because the distal gastric mucosa is a popular site for the development of intestinal metaplasia and subsequently of cancer with the differentiated type.

Previous studies showed that the patients with SMEGC have a higher rate of a family history of gastric cancer, and smoking and drinking may also be related to the occurrence of SMEGC [11, 12]. The results of a study [5] in South Korea showed that a family history of gastric cancer in patients with SMEGC was more common than that in patients with single EGC (27.4% vs. 16.4%; *P* = 0.002). In another study, Isobe et al. [3] compared 146 multiple gastric cancer cases and 1194 single gastric cancer cases and found that older men who smoked and drank and had a family history of gastric cancer were at high risk for SMEGC. Their analysis also suggests that genetic and environmental conditions may be associated with the familial aggregation of multiple gastric cancers. However, the impact of smoking and drinking on SMEGC remains controversial. Even in the studies of Isobe et al., multivariate analysis did not find that smoking or drinking was an independent risk factor for predicting SMEGC. In our study, the results showed that the rates of family history of gastric cancer and drinking habits in SMEGC patients were similar to those in the patients with single EGC. Although the rate of patients with smoking habits in SMEGC group was higher (48.6% vs. 34.2%), the difference was not statistically significant.

As is well known, *H. pylori* infection is associated with gastric cancer. Some studies have suggested that *H. pylori* eradication therapy has prophylactic power against metachronous gastric cancer, which develops more than 1 year after the primary lesion treatment [13, 14]. However, most studies have shown that *H. pylori* infection does not increase the risk of SMEGC, including the newly discovered lesion at another site in the stomach within 1 year after resection for the primary lesion. The study by Lim et al. [10] even found that *H. pylori* infection was less in SMEGC patients than in isolated EGC patients (56.2% and 50.9%, *p* = 0.011). One of the possibilities is that SMEGC tends to occur in elderly people with gastric atrophy and intestinal metaplasia. These people often have lower rates of *H. pylori* infection because of the poor stomach environment for *H. pylori*. In our study, 8 patients (21.6%) in the multiple groups had evidence of *H. pylori* infection, which was lower than that in the single group, but the difference was not statistically significant.

SMEGC lesions are more likely to be overlooked during endoscopy, which may be associated with the endoscopic 'doctors' knowledge and experience, the quality and proficiency of endoscopy, the site of the lesions, the morphological characteristics of the lesions, etc. A study indicates that even expert endoscopists can miss minor lesions in as many as 27.5% of patients with SMEGC [15]. Some studies have reported that small lesions and flat lesion types are major risk factors for endoscopic failure in recognizing additional lesions [15–17]. Lee et al. [15] compared 16 missed lesions and 42 detected lesions in multiple gastric cancer, and the results showed that the diameter of the missed lesions was significantly smaller than that of the detected lesions (1.57 ± 0.74 cm vs. 2.14 ± 1.40 cm, *P* = 0.005). In this study, a total of 17 lesions in 16 cases were missed before ESD, the missed lesions were more likely to be flat and depressed (*P* < 0.05), and the diameter of the missed lesions was significantly smaller than that detected by preoperative endoscopy (0.69 ± 0.43 cm vs. 1.30 ± 1.21 cm, *P* < 0.05). In addition, a multicenter retrospective cohort study [18] showed that 21 of the 110 SMEGCs (19%) were missed at the time of initial ESD, and indicated that many of the missed lesions were located in the upper third of the stomach, and the missed rate was associated with the 'endoscopist's inexperience (<500 esophago-gastro-duodenoscopy cases).

Considering the incidence and missed diagnosis rate of SMEGC, it is important to adopt some measures to improve the detection rate of multiple lesions. Studies have shown that increased preoperative endoscopic screening time helps to reduce missed diagnoses. Lee et al. [19] compared patients in whom multiple lesions were completely diagnosed before resection (complete examination group) and those in whom multiple lesions were not fully diagnosed prior to resection, but were found during the follow-up evaluation (incomplete examination group), and found that the entire examination group had a significantly longer examination time than the incomplete examination group (6.5 ± 2.4 min and 3.8 ± 1.8 min, *P* < 0.001). In our study, we performed total gastric endoscopy for EGC patients again before the operation during the process of ESD. We found that the preoperative detection rate of SMEGC was 56.8%, the detection rate of intraoperative endoscopic screening was 24.3% (9/37), and that of postoperative follow-up within 1 year was 18.9%. The missed diagnosis rate of SMEGC was as high as 43.2% before ESD. When we performed endoscopic screening during ESD operation, there was a 24.3% increase in the overall diagnosis rate of SMEGC. Therefore, in addition to confirming the detected lesions before ESD, it is necessary to re-examine the entire stomach during ESD operation, which can reduce the missed diagnosis of multiple lesions.

There were some limitations in this study. First, all cases included in the study were from one single-center. Second, all the patients with EGC were the cases treated with ESD. Third, we did not analyze some factors that might affect the quality of endoscopy, such as the duration of endoscopy and the experience of different endoscopists.

In conclusion, SMEGC lesions can be often overlooked endoscopically. Therefore, more careful endoscopic observations are needed in detecting SMEGC, especially in elderly patients or (and) patients with severe atrophic gastritis during endoscopic screening. Endoscopic screening during ESD operation can effectively reduce the missed diagnosis rate of SMEGC.

## Figures and Tables

**Table 1:** Predictive factors of SMEGC compared with solitary EGC.

	SMEGC (*n* = 37)	Single EGC (*n* = 234)	Univariate *P* value
Age, years			
<60	6 (15.8%)	83 (35.5%)	0.02
≥60	31 (83.8%)	151 (64.5%)	
Gender			
Male	28 (75.7%)	152 (65.0%)	0.2
Female	9 (24.3%)	82 (35.10)	
Family history of gastric cancer			
No	34 (92.1%)	212 (90.6%)	0.801
Yes	3 (7.9%)	22 (9.4%)	
Drinking habits			
Non-drinker	26 (70.3%)	168 (71.8%)	0.847
Occasional-drinker	7 (18.9%)	36 (15.4%)	
Daily-drinker	4 (10.8%)	30 (12.8%)	
Smoking habit			
Nonsmokers	19 (51.4%)	154 (65.8%)	0.190
Light smokers	5 (13.5%)	19 (8.1%)	
Heavy smokers	13 (35.1%)	61 (26.1%)	
Severe atrophic gastritis			
No	19 (51.4%)	163 (69.7%)	0.028
Yes	18 (48.6%)	71 (30.3%)	
*H. pylori* infection			
No	29 (78.4%)	173 (73.9%)	0.564
Yes	8 (21.6%)	61 (26.1%)	
Histology			
Differentiated type	35 (94.6%)	218 (93.2%)	0.745
Undifferentiated type	2 (5.4%)	16 (6.8%)	
Invasion depth			
M	36 (94.6%)	223 (95.3%)	0.975
SM1	1 (2.7%)	5 (2.1%)	
SM2	1 (2.7%)	6 (2.6%)	
LV invasion			0.438
Negative	36 (97.3%)	231 (98.7%)	
Positive	1 (2.7%)	3 (1.3%)	

M, confined to mucosa; SM1, depth of invasion from the muscularis mucosa <500 *μ*m; SM2, depth of invasion from the muscularis mucosa ≥500 *μ*m; LV, lymphovascular.

**Table 2:** Multivariate analysis of risk factors for SMEGC.

	OR	95% CI	*P* value
Age, years			
≥60	2.63	1.036–6.680	0.042
Gender			
Male	0.831	0.361–2.269	0.905
Smoking habit			
Nonsmokers	1		
Light smokers	1.904	0.595–6.091	0.278
Heavy smokers	1.640	0.687–3.913	0.265
Severe atrophic gastritis			
Yes	1.906	0.919–3.951	0.083

**Table 3:** SMEGC lesion diagnosed before ESD (*n* = 21).

No.	Gender	Age, years	Main lesions				Minor lesions			
			Location	Macroscopic classification	Size (cm)	Depth of invasion	Location	Macroscopic classification	Size (cm)	Depth of invasion
1	Male	59	Middle	IIa + IIc	1.0	M	Lower∗2	IIa/IIa	0.6/0.4	M
2	Male	62	Lower	IIc	2.0	M	Upper	IIc	0.8	M
3	Male	60	Lower	IIa	1.6	M	Upper	IIa + IIc	1.0	M
4	Female	60	Middle	IIa	1.5	M	Lower	IIc	1.0	M
5	Female	70	Lower	IIa + IIc	1.5	M	Lower	IIc	1.2	M
6	Female	72	Lower	IIc	0.8	M	Lower	IIa	0.6	M
7	Male	72	Middle	IIa	1.5	M	Middle	IIc	0.8	M
8	Male	59	Middle	IIa + IIc	1.0	M	Lower	IIc	0.7	M
9	Male	61	Upper	IIa	1.2	M	Lower	IIc	0.8	M
10	Male	66	Upper	IIa + IIc	1.0	Sm2	Upper	IIa	0.5	M
11	Male	69	Upper	IIa + IIc	1.0	M	Upper	IIb	0.6	M
12	Female	73	Middle	IIa + IIc	1.0	M	Lower	IIc	0.7	M
13	Male	63	Lower	IIc + III	4.0	Sm1	Lower	IIb	1.0	M
14	Female	72	Middle	IIa + IIc	1.5	M	Lower	IIc	0.6	M
15	Male	52	Lower	IIc	0.5	M	Lower	IIc	0.4	M
16	Male	66	Lower	IIc	1.6	M	Upper	IIc	0.6	M
17	Female	61	Lower	IIa + IIc	1.0	M	Lower	IIc	0.6	M
18	Male	62	Lower	IIa + IIc	3.0	M	Lower	IIa	0.5	M
19	Male	67	Middle	IIa	1.5	M	Middle	IIa	0.5	M
20	Male	52	Lower	IIc	0.6	M	Lower∗2	IIc	0.5/0.4	M
21	Male	61	Lower	IIc	1.0	M	Lower	IIc	0.7	M

∗ means the number of the lesion.

**Table 4:** SMEGC lesion diagnosed during ESD operation (*n* = 9).

No.	Gender	Age, years	Diagnosed before ESD				Diagnosed during ESD operation			
			Location	Macroscopic classification	Size (cm)	Depth of invasion	Location	Macroscopic classification	Size (cm)	Depth of invasion
1	Male	68	Lower	IIa	2.5	M	Lower	IIa	0.5	M
2	Male	62	Middle	IIc	1	M	Middle	IIc	0.5	M
3	Male	69	Middle	IIa	1.5	M	Lower	IIc	0.5	M
4	Female	62	Middle	IIa + IIc	0.7	M	Lower	IIc	0.4	M
5	Male	68	Lower	IIa	1.5	M	Lower	IIc	0.8	M
6	Male	76	Lower	IIa	5	M	Lower	IIa	0.6	M
7	Male	68	Middle	IIa	2	M	Middle	IIc	0.5	M
8	Male	42	Lower∗2	IIc/IIb	1.0/0.7	M	Lower	IIb	0.4	M
9	Male	64	Lower	IIc	2.5	M	Middle	IIc	1.0	M

**Table 5:** SMEGC lesion diagnosed during follow-up within 1 year (*n* = 7).

No.	Gender	Age, years	Diagnosed before ESD				Diagnosed during follow-up			
			Location	Macroscopic classification	Size (cm)	Depth of invasion	Location	Macroscopic classification	Size (cm)	Depth of invasion
1	Male	66	Lower	IIa	1	M	Middle	IIb	2	M
2	Male	57	Middle	IIa	8	M	Lower	IIc	0.5	M
3	Male	69	Middle	IIa	0.8	M	Middle	IIa	0.5	M
4	Male	59	Lower	IIa	1	M	Upper∗2	IIb/IIb	0.5/0.6	M
5	Female	56	Middle	IIa	1	M	Middle	IIa	0.6	M
6	Male	70	Middle	IIa + IIb	1.8	M	Upper	IIb	1.5	M
7	Female	72	Middle	IIa + IIc	1	M	Lower	IIc	0.5	M

∗ means the number of the lesion.

**Table 6:** The clinicopathologic features between missed lesions and detected lesions.

	Missed lesions (*n* = 17)	Detected lesions (*n* = 61)	Univariate *P* value
Size (cm)	0.69 ± 0.43	1.30 ± 1.21	0.047
Location			
Upper	3 (17.7%)	8 (13.1%)	0.861
Middle	5 (29.4%)	17 (27.9)	
Lower	9 (52.9%)	36 (59.0%)	
Macroscopic classification			
Elevated	4 (23.5%)	33 (54.1%)	0.009
Flat	5 (23.5%)	2 (3.3%)	
Depressed	8 (53.0%)	26 (42.6%)	
Histology			
Differentiated type	17 (100%)	58 (95.1%)	0.591
Undifferentiated type	0 (0%)	3 (4.9%)	
Invasion depth			
M	17 (100%)	59 (96.7)	0.751
SM1	0 (0%)	1 (1.6%)	
SM2	0 (0%)	1 (1.6%)	

## Data Availability

The data used to support the findings of this study are included within the article.
